# Promyelocytic Blastic Crisis in Chronic Myeloid Leukemia During Imatinib Treatment

**DOI:** 10.4274/tjh.2014.0211

**Published:** 2015-05-08

**Authors:** Federico Angriman, Maria Nelly Gutierrez Acevedo, Maria Sol Rossi, Alberto Daniel Gimenez Conca, Victoria Otero, Jorge Alberto Arbelbide, Hernán Michelángelo

**Affiliations:** 1 Buenos Aires University School of Medicine, Hospital Italiano de Buenos Aires, Department of Internal Medicine, Buenos Aires, Argentina; 2 Hospital Italiano de Buenos Aires, Department of Internal Medicine, Buenos Aires, Argentina; 3 Hospital Italiano de Buenos Aires, Department of Internal Medicine, Division of Hematology, Buenos Aires, Argentina

**Keywords:** Acute promyelocytic leukemia, All-trans retinoic acid, BCR/ABL, PML/RAR-α, Imatinib

An 82-year-old woman was admitted to our hospital presenting with febrile neutropenia. She had been diagnosed with chronic myeloid leukemia 2 years ago and had been on imatinib treatment since [1]. A month before admission she presented with malaise, anemia, and mild leukopenia; a bone marrow aspirate and biopsy performed 20 days before admission showed no alterations. Imatinib dosing was adjusted but mild cytopenia persisted. The patient presented with acute abdominal pain, fever, and shaking chills to the emergency department. On physical examination the patient was awake and appeared uncomfortable. She had pain in the left lower abdominal quadrant. Petechiae were evident on the lower limbs. Complete blood count revealed anemia, severe neutropenia, and thrombocytopenia (Table 1). Overt disseminated intravascular coagulation was present. Abdominal computed tomography showed acute diverticulitis. The patient was started on broad-spectrum antibiotics. Informed consent was obtained.

A peripheral blood smear revealed more than 30% circulating promyelocytic blasts. A bone marrow aspirate and biopsy showed hypercellular marrow with myeloid hyperplasia and more than 90% myeloblasts (Figure 1). The blasts displayed hypergranular cytoplasm with bundles of Auer rods. PML/RAR-α was positive according to real-time PCR of the bone marrow. A diagnosis of promyelocytic blastic crisis was made [2,3,4,5]. 

## Figures and Tables

**Table 1:**
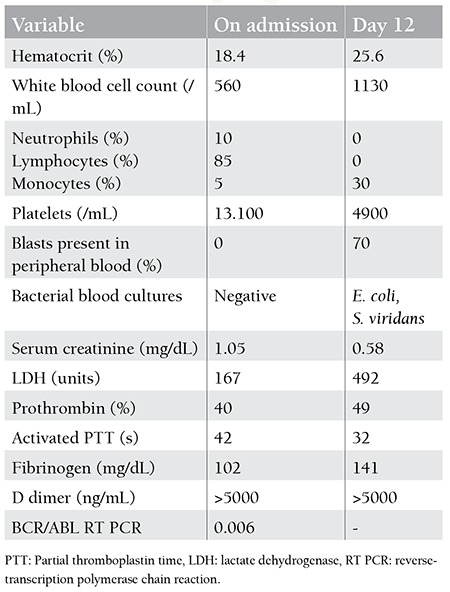
Results of laboratory testing.

**Figure 1:**
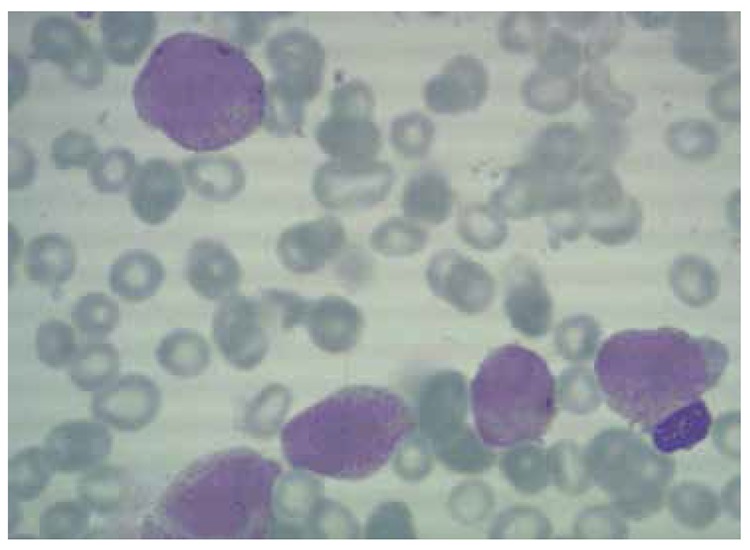
Leukemic cell morphology of bone marrow aspiration specimen (May-Grünwald-Giemsa).

## References

[1] Faderl S, Talpaz M, Estrov Z, O’Brien S, Kurzrock R, Kantarjian HM (1999). The biology of chronic myeloid leukemia. N Engl J Med.

[2] Calabretta B, Perrotti D (2004). The biology of CML blast crisis. Blood.

[3] Shichishima T, Abe R, Satoh T, Kawaguchi M, Uchida T, Kariyone S (1987). Promyelocytic crisis of chronic myelocytic leukemia: case report and review of the literature. Nihon Ketsueki Gakkai Zasshi.

[4] Abe R, Shichishima T, Kawaguchi M, Uchida T, Kariyone S (1986). Promyelocytic crisis of chronic myelogenous leukemia without t(15;17). Cancer Genet Cytogenet.

[5] Gozzetti A, Bocchia M, Calabrese S, Pirrotta MT, Crupi R, Raspadori D, Lauria F (2007). Promyelocytic blast crisis of chronic myelogenous leukemia during imatinib treatment. Acta Haematol.

